# Consumption of pharmaceutical drugs in exception region of separation for drug prescribing and dispensing program in South Korea

**DOI:** 10.1186/s13011-015-0032-3

**Published:** 2015-09-16

**Authors:** Sang Mi Yuk, Kyu-Tae Han, Sun Jung Kim, Woorim Kim, Tae Yong Sohn, Byungyool Jeon, Young-Man Kim, Eun-Cheol Park

**Affiliations:** Department of Health Policy and Management, Graduate School of Public Health, Yonsei University, Seoul, Republic of Korea; Health Insurance Review and Assessment Service, Seoul, Republic of Korea; Department of Public Health, Graduate School, Yonsei University, Seoul, Republic of Korea; Institute of Health Services Research, Yonsei University College of Medicine, Seoul, Republic of Korea; Department of Health Administration and Management, College of Medical Science, Soonchunhyang University, Asan, Korea; Department of Health Services Administration, Yuhan University, Bucheon, Republic of Korea; Department of Preventive Medicine, CHA University, Pochon, Republic of Korea; Gyeonggi Infectious Disease Control Center, Seongnam, Republic of Korea; Department of Preventive Medicine, Yonsei University College of Medicine, Seoul, Republic of Korea

**Keywords:** Separation of drug prescribing and dispensing, Pharmaceutical reform, Pharmaceutical expenditures, Misuse

## Abstract

**Background:**

In the year 2000, the South Korean government introduced a program for separation of drug prescribing and dispensing. The goals of the program are to reduce misuse of drugs and to contain drug expenditures. The government also designated exception regions for the program to reduce the inconvenience for people who reside in areas with a shortage of health care resources. However, according to government reports, many adverse events related to drug misuse occurred in these exception regions after the program reforms were introduced. Therefore, it is worth investigating the factors that relate to drug consumption so that misuse in exception regions can be reduced.

**Methods:**

Data from medical institutions, detailed drug supply data, and community health survey data were included in the analysis. Multilevel linear regression analysis using mixed models that included pharmacy—and regional-level variables were used to examine the associations regarding the percentages of drug types consumed (i.e., antipyretic, analgesic, anti-inflammatory drugs, psychotropic drugs, adrenal cortical hormones, and antibiotics).

**Results:**

The data used in this analysis were from a total of 16,455 pharmacies. There were 1.9 % pharmacies from program exception regions and 98.1 % pharmacies from program application regions. Compared with the pharmacies in the program application regions, the exception region pharmacies had higher values for percent consumption of the antipyretic, analgesic, anti-inflammatory drugs category, and of the adrenal cortical hormones category (antipyretic, analgesic, anti-inflammatory drugs = β: 3.19, Standard Error (SE): 0.82, t: 3.88, *p*-value < 0.05; adrenal cortical hormones = β: 0.72, SE: 0.07, t: 9.92, *p*-value < 0.05).

**Conclusion:**

Our results suggested that pharmacies in exception regions supplied more antipyretic, analgesic, anti-inflammatory drugs, and more adrenal cortical hormones compared with the pharmacies where separation of drug prescribing and dispensing had been implemented. Health care professionals and health policy makers should consider management of health care expenditure by the category of drugs consumed, especially in program exception regions.

## Background

South Korea has experienced gradual socio-economic development since the 1980s. This development has resulted in increased health care accessibility and demand, and the consumption of medical care by South Koreans has gradually increased. Individuals have easier access to medical resources compared with the past, and the consumption of general pharmaceutical products has increased [1]. The overall health status of the population has improved, but new problems (e.g., increasing medical costs) have developed. Solutions to these problems are needed [2].

In the year 2000, the Korean government introduced a program reform that separated the prescribing and dispensing of pharmaceutical drugs. The objectives of the reform were to reduce misuse of drugs, and to contain drug expenditures. Before the program was introduced, individual physicians and pharmacists could both prescribe and dispense drugs, which was a system that resulted in an inefficient treatment for the patient. However, application of the program reform to all of South Korea was difficult because of the presence of regions with shortages of health care resources [3, 4]. To reduce the inconvenience of populations residing in these areas, the South Korean government designated these regions as exceptions to the separation of prescribing and dispensing rule. It was expected that reforming the program would result in a more efficient management of consumption and reduced pharmaceutical expenditures [5].

Contrary to government expectations, pharmaceutical expenditures were not well-controlled after the program reform was introduced. Organization for Economic Co-operation and Development (OECD) health data expenditure trend analyses results indicate that compared with other OECD countries, expenditure for pharmaceuticals in South Korea has gradually increased (Y2000 = average for OECD countries: 1.3 % of Gross Domestic Product (GDP), South Korea: 1.0 % of GDP; Y2005 = average for OECD countries: 1.4 % of GDP, South Korea: 1.3 % of GDP; Y2010 = average for OECD countries: 1.4 % of GDP, South Korea: 1.6 % of GDP) [6].

To investigate the factors associated with this problem, many health care professional have examined factors associated with the separation of drug prescribing and dispensing [7–9]. Few studies examining the effects of the exception regions have been published [10]. The South Korean government reported that many adverse events related to drug misuse have occurred in the exception regions since the program reforms were introduced [11, 12]. Under the reformed pharmaceutical affairs act, pharmacist with pharmacy in exception regions for separation of drug prescribing and dispensing could prescribe medication to patients without a doctor’s prescription. Therefore, the monitoring for providing health care was not well managed compared to those in the application region. For that reason, it might easily cause many adverse events related to misuse. Nevertheless, there were no evidence-based studies about the factors associated with pharmaceutical expenditures to reconsider the program for separation of drug prescribing and dispensing. Hence to solve that problem, the aim of our study was to analyze the factors associated with drug consumption in the exception regions compared with the application regions.

## Methods

### Sampled data

**Fig. 1:**
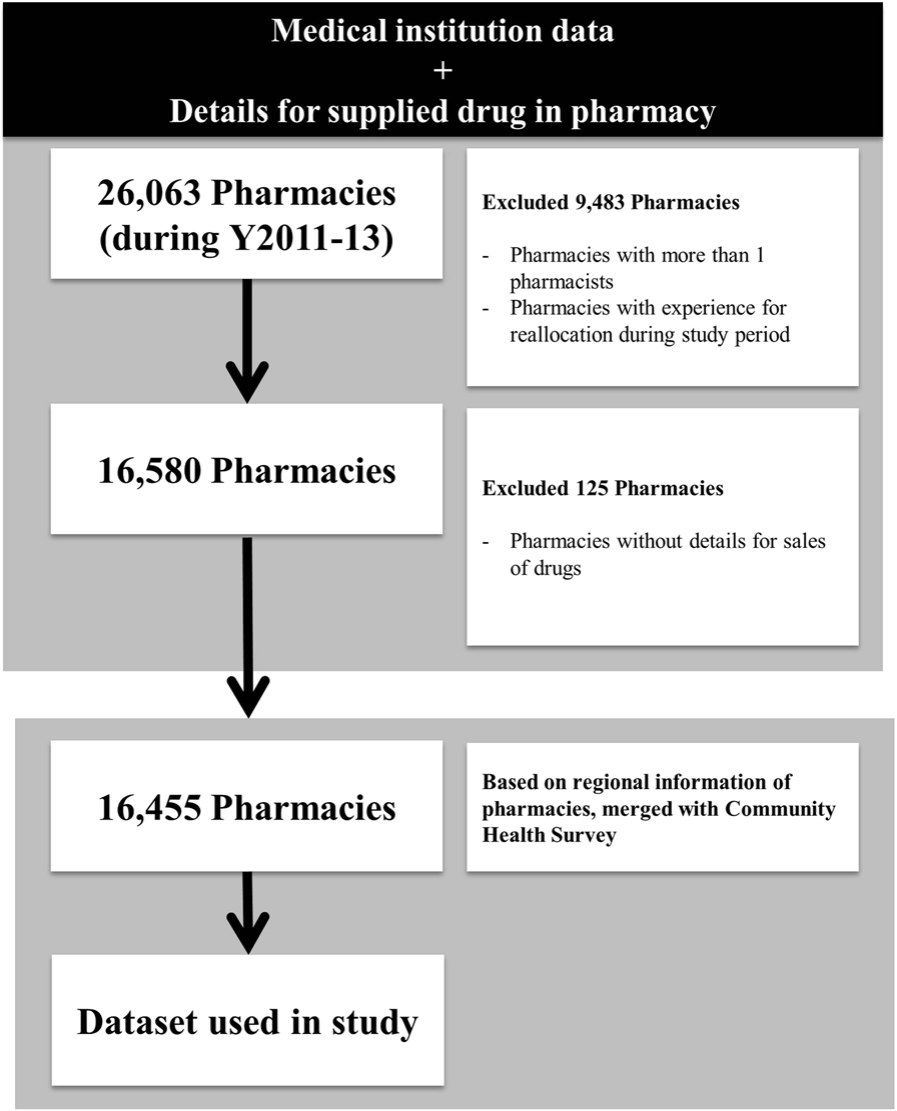
Sampling method for data used in this study

Three types of data were combined for analysis. We first sampled the study pharmacies included in the medical institution data of the Health Insurance Review & Assessment Service. Next, to analyze drug consumption details and to examine the regional characteristics of each pharmacy, we merged drug supply details from the Korea Pharmaceutical Information Service Center and the community health survey of the Korea Centers for Diseases Control & Prevention with sampling data from medical institutions. These data were collected between 2011 and 2013 and included 26,063 pharmacies. We excluded data from pharmacies with more than one pharmacist, pharmacies that relocated during the study period, and pharmacies that did not supply financial details of the supplied drugs, from the analysis. Finally, the data used in this study consisted of 16,455 pharmacies in 247 regions (Fig. 1).

### Variables

The outcome variable was percent drug consumption of drugs including antipyretics, analgesics, anti-inflammatories, psychotropic drugs, adrenal cortical hormones, and antibiotics, of the total drug consumption for each pharmacy. This variable was defined as the ratio of the sum of pharmacy costs for specific drug purchase to the total pharmacy costs for drug purchase in each pharmacy. The percentages were calculated as:

$$ \begin{array}{l} Percentage\  of\  drug\  consumption\\ {} = \left( sum\  of\  pharmacy\  costs\ for\  specific\  drug\ /\  total\  pharmacy\  costs\ for\  drug\  purchase\right)\kern0.62em x\kern0.75em 100\end{array} $$

The primary variable of interest was exception from/application to the separation of drug prescribing and dispensing program. Whether a pharmacy was in an exception region was defined using the criteria established by the program policy decision makers:

1. Island region or rural region without a medical institution
2. Region with a pharmacy, designated as an exception region by decision makers
3. Region where distance between medical institution and pharmacy was >1 km
4. Region within a military installation security zone or within a limited development district

We adjusted for pharmacy-level and regional-level variables when we analyzed the relationships between program designation (i.e., exception region or application region) and drug consumption rate. Pharmacy-level variables encompassed both pharmacist and pharmacy characteristics: Sex of pharmacist, Age of Pharmacist, period of time pharmacy had been in business, whether separation of drug prescribing and dispensing occurred, time period that the program applied, and total drug purchase amount. Age-groups were categorized as <45 years, 46–55 years, 56–65 years, and ≥66 years. The period of time the pharmacy had been in business was defined as the time since a pharmacy first began operation. This variable was categorized as <12 months, 13–24 months, and ≥25 months. The time period designated as an exception region was defined as the period during which the program exception criteria was applied. The total pharmacy costs for drug purchase were calculated as the total amount spent to supply the drugs in each pharmacy during the study period. The characteristics of the regions with the pharmacies were as follows: name of region, total number of clinics in the region, total number of pharmacies in the region, mean individual income in the region, and the proportion of national basic livelihood security beneficiaries in the region. Total number of clinics/pharmacies in the region was categorized by each average value. In addition, the proportion of national basic livelihood security beneficiaries in a region was calculated as the number of national basic livelihood security beneficiaries divided by the total population size.

### Statistical analysis

We first analyzed the general characteristics of each group by examining the distributions of each variable. We performed *χ*^2^ tests and Mann–Whitney U tests to examine differences in each variable by program designation. Next, we performed Mann–Whitney U tests and Kruskal-Wallis tests to compare the average values and standard deviations for the percentage of specific drug consumption with the independent variables, because dependent variables in this study were continuous and did not have a normal distribution.

Third, multilevel linear regression analysis using mixed models that included pharmacy—and regional-level variables were utilized to examine the associations between program designation (i.e., exception region or application region) and percentages of drug consumption (antipyretic, analgesic, anti-inflammatory drugs, and psychotropic drugs, adrenal cortical hormones, and antibiotics) in hierarchical data which was consisted of pharmacy and regional levels [13, 14].

In using such methodology, we compared model specification by each model (model 1 = empty model, model 2 = only adjusted pharmacist and pharmacy level, model 3 = only adjusted regional level, and model 4 = fully adjusted). We also compared intra-class correlation coefficient (ICC) to examine the reliability of each level variable for the outcome variable. The ICC was defined as the ratio of the between cluster variance to the total variance. It was interpreted through correlation among observations within the same cluster.

Finally, we performed sub-group analysis for multilevel linear regression analysis in the exception regions with respect to pharmacist (age and sex) and pharmacy (region and time since open for business) characteristics. All analyses were performed using SAS software (ver. 9.2, Cary, North Carolina, USA). A *P*-value <0.05 was considered to indicate a statistically significant result.

## Results

A total of 16,455 pharmacies were included in the analysis. There were 1.9 % pharmacies in the exception regions and 98.1 % pharmacies in the program application regions.

Table 1 presents the results of the univariate analyses between the independent variables and the program designation. Based on the results of the Chi-square test for categorical variables, the exception regions had more male pharmacists than the application regions (X^2^ = 62.6, degrees of freedom; df = 1, *P <* .0001). Exception regions also had higher percentages of elderly pharmacists (X^2^ = 168.1, df = 3, *P <* .0001). Compared with the program application pharmacies, a higher percentage of the program exception pharmacies were located in non-metropolitan areas (X^2^ = 254.9, df = 1, *P <* .0001). There were higher numbers of clinics/pharmacies in the program application regions. In the results of Mann–Whitney *U* test for comparing the averages and standard deviations in continuous variables, it can be seen that the average values of pharmacy total drug purchase amount was lower in exception region pharmacies (Z = −18.7, *P <* .0001). The average values for individual income was also higher in program application regions (Z = 3.7, *P =* 0.0002) (Table 1).

**Table 1:** General characteristics of pharmacists and pharmacies

Variables	Separation of drug prescribing and dispensing (N=16,455)				Test statistics (df)	*P*-Value
	Exception		Application			
	N/Mean	%/SD	N/Mean	%/SD		
Pharmacy characteristics						
Sex of pharmacist						
Male	224	72.5	8,036	49.8	*X* ^*2*^(1)=62.6	
Female	85	27.5	8,110	50.2		
Age of pharmacist (years)						
≤45	27	8.7	4,890	30.3	*X* ^*2*^(3)=168.1	<.0001^a^
46–55	51	16.5	4,367	27.1		
56–65	74	24	3,268	20.2		
≥66	157	50.8	3,621	22.4		
Length of Operation						
≤12 months	48	15.5	2,329	14.4	*X* ^*2*^(2)=5.4	0.067^a^
13–24 months	49	15.9	1,914	11.9		
≥25 months	212	68.6	11,903	73.7		
Period of exclusion for reformed program						
≤18 months	95	30.7	16,146	100	*X* ^*2*^(1)=11329.4	<.0001^a^
≥19 months	214	69.3	0	0		
Total pharmacy cost for drug purchase (10 million KRW)	18.0	±19.1	75.0	±75.2	Z=−18.7	<.0001^b^
Regional Characteristics						
Region						
Metropolitan (N=74)	14	4.5	8,134	50.4	*X* ^*2*^(1)=254.9	<.0001^a^
Non-metropolitan (N=173)	295	95.5	8,012	49.6		
Total number of clinics in regions with pharmacies						
≤60 (N=212)	304	98.4	11,961	74.1	*X* ^*2*^(1)=94.3	
≥61 (N=35)	5	1.6	4,185	25.9		
Total number of pharmacies in regions with pharmacies						
≤45 (N=176)	273	88.4	10,606	65.7	*X* ^*2*^(1)=69.5	
≥46 (N=71)	36	11.7	5,540	34.3		
Average per capita income in regions with pharmacies						
≤38 million KRW (N=175)	232	75.1	8,442	52.3	*X* ^*2*^(1)=63.2	
≥39 million KRW (N=72)	77	24.9	7,704	47.7		
Proportion of national basic livelihood security beneficiaries in regions with pharmacies	3.2	±1.6	2.9	±1.5	Z=3.7	0.0002^b^
Total	309	1.9	16,146	98.1		

Note. Significant level *P <* 0.05. If these values were lower than 0.05, it indicated that the distribution or mean/standard deviation of each independent variable were differenced by separation of drug prescribing and dispensing. *KRW* Republic of Korea Won, *df* degrees of freedom

^a^P for Chi-square test, Chi-square tests were used to examine the differences in distribution of each categorical variable by separation of drug prescribing and dispensing

^b^p for Mann–Whitney *U* test, Mann–Whitney U tests were used to examine differences in mean/standard deviation of each continuous variable by separation of drug prescribing and dispensing

Table 2 presents the results for Mann–Whitney *U* test and Kruskal-Wallis test to compare average values and standard deviation regarding the percentages of specific drugs consumed by each independent variable. Based on the results of Mann–Whitney U test, the average percentages of antipyretic, analgesic, anti-inflammatory drugs, and adrenal cortical hormones were higher in the exception regions compared to the application regions (antipyretic, analgesic, anti-inflammatory drugs = Z: 18.5, *P <* .0001; adrenal cortical hormones = Z: 14.0, *P <* .0001). The average percentages of those 2 types of drugs consumed were higher for pharmacies that had been operating in an exception region for a longer period of time (antipyretic, analgesic, anti-inflammatory drugs = Z: 16.8, *P <* .0001; adrenal cortical hormones = Z: 12.2, *P <* .0001). In the results of the Kruskal-Wallis test, the average of drugs consumed for psychotropic drugs, adrenal cortical hormones, and antibiotics were higher in pharmacies which were in operation for a shorter time (psychotropic drugs = X^2^: 8.2, df: 2, *P =* 0.0109; adrenal cortical hormones = X^2^: 18.4, df: 2, *P <* .0001; antibiotics = X^2^: 173.7, df: 2, *P <* .0001) (Table 2).

**Table 2:** Percentages of drug consumption categorized by pharmacist and pharmacy variables, by four drug categories

Variables	Antipyretic; Analgesic;				Psychotropic drugs				Adrenal cortical hormones				Antibiotics			
	Anti-inflammatory drugs															
	Mean	SD	Test statistics (df)	*P*-Value	Mean	SD	Test statistics (df)	*P*-Value	Mean	SD	Test statistics (df)	*P*-Value	Mean	SD	Test statistics (df)	*P*-Value
Pharmacy characteristics																
Sex of pharmacist																
Male	9.86	9.46	Z = −11.6574	<.0001†	0.82	2.35	Z = 4.6	<.0001†	0.29	0.71	Z = −3.3	0.0021†	6.77	30.87	Z = 7.9	<.0001†
Female	9.03	6.46			0.91	2.47			0.28	0.68			7.24	8.71		
Age of pharmacist (years)																
≤45	9.20	11.17	X^2^(3) = 382.8	<.0001^a^	0.95	2.63	X^2^(3) = 207.2	<.0001^a^	0.32	0.73	X^2^(3) = 85.1	<.0001^a^	8.90	32.82	X^2^(3) = 378.3	<.0001^a^
46–55	8.72	5.99			0.98	2.35			0.26	0.55			7.00	7.96		
56–65	9.30	5.88			0.89	2.68			0.26	0.60			6.01	9.07		
≥66	10.75	7.04			0.60	1.83			0.30	0.87			5.44	26.32		
Length of operation																
≤12 months	9.94	15.77	X^2^(2) = 1.4	0.3231^a^	0.92	2.67	X^2^(2) = 8.2	0.0109^a^	0.35	0.96	X^2^(2) = 18.4	<.0001^a^	10.45	56.56	X^2^(2) = 173.7	<.0001^a^
13–24 months	9.64	6.25			0.86	2.02			0.33	0.78			7.99	8.58		
≤25 months	9.32	5.86			0.86	2.41			0.27	0.62			6.17	7.66		
Separation of drug prescribing and dispensing																
Exception	15.21	6.31	Z = 18.5	<.0001†	0.39	1.08	Z = −15.2	<.0001†	1.09	1.55	Z = 14.0	<.0001†	6.01	8.81	Z = 2.3	0.0232†
Application	9.34	8.11			0.88	2.42			0.27	0.66			7.03	22.91		
Period of exclusion for reformed program																
≤18 months	9.36	8.11	Z = 16.8	<.0001†	0.87	2.42	Z = −14.1	<.0001†	0.28	0.67	Z = 12.2	<.0001†	7.02	22.87	Z = 2.8	0.0057†
≥19 months	15.69	5.63			0.34	1.12			1.10	1.57			5.76	3.61		
Regional characteristics																
Region																
Metropolitan	9.12	6.23	Z = −10.5	<.0001†	0.86	2.60	Z = −8.1	<.0001†	0.26	0.63	Z = −11.3	<.0001†	7.00	19.34	Z = −2.0	0.0404†
Non-metropolitan	9.77	9.61			0.88	2.20			0.32	0.76			7.02	25.61		
Total number of clinics in regions with pharmacies																
≤60	9.53	6.19	Z = −7.5	<.0001†	0.86	2.44	Z = −3.1	0.0020†	0.29	0.71	Z = −6.5	<.0001†	6.99	16.42	Z = −3.7	0.†
≥61	9.21	12.1			0.89	2.29			0.27	0.66			7.05	35.20		
Total number of pharmacies in region with pharmacies																
≤45	9.45	6.20	Z = −2.2	0.0296†	0.88	2.57	Z = 0.2	0.8213†	0.30	0.69	Z = −9.2	<.0001†	7.30	16.90	Z = −13.3	<.0001†
≥46	9.45	10.93			0.85	2.06			0.26	0.71			6.44	31.08		
Average per capita income in regions with pharmacies																
≤38 million KRW	9.77	9.48	Z = −9.2	<.0001†	0.87	2.22	Z = −9.6	<.0001†	0.29	0.69	Z = −4.5	<.0001†	6.55	25.65	Z = 11.3	<.0001†
≥39 million KRW	9.09	6.23			0.87	2.59			0.28	0.71			7.52	18.92		
Total	9.40	8.10			0.90	2.40			0.30	0.70			7.00	22.70		

Note. Significant level *P <* 0.05. If these values were lower than 0.05, it indicated that the mean/standard deviation of drug consumption were differenced by each independent variable. *KRW* Republic of Korea Won, *df* degrees of freedom

†p for Mann–Whitney *U* test, Mann–Whitney U tests were used to examine differences in mean/standard deviation of drug consumption by each categorical variable as these did not have normal distribution and below than 3 groups

^a^p for Kruskal-Wallis test, Kruskal-Wallis tests were used to examine differences in mean/standard deviation of drug consumption by each categorical variable as these did not have normal distribution and above than 3 groups

Tables 3, 4, 5 and 6 presents the results of the multilevel regression analysis using mixed model for adjusting pharmacy—and regional-level analyses. In the results for antipyretic, analgesic, anti-inflammatory drugs, pharmacies with male pharmacists and elderly pharmacists were more likely to dispense antipyretic, analgesic, anti-inflammatory drugs. Pharmacies that had been in operation for a longer time period were inversely associated with the consumption of antipyretic, analgesic, anti-inflammatory drugs. The total pharmacy costs for drug purchase, which is an indirect indicator for the size of each pharmacy, were inversely related to the percentages of antipyretic, analgesic, anti-inflammatory drugs consumed. The pharmacies in exception regions had higher values for percentages of antipyretic, analgesic, anti-inflammatory drugs dispensed compared to pharmacies in the program application regions (β: 3.19, Standard Error (SE): 0.82, t: 3.88, df: 93, *p*-value < 0.05). By the results of the regional-level variables, pharmacies which were located in metropolitan regions had inverse associations with less drug consumption. The region with higher individual income also had such relationships. A higher proportion of national basic livelihood security beneficiaries had higher consumption percentages of drugs in the antipyretic, analgesic, anti-inflammatory drug category (Table 3). In the results regarding psychotropic drugs, pharmacies with younger pharmacists or with shorter lengths of operation were more likely to dispense psychotropic drugs. However, there were no significant relationships with the separation of drug prescribing and dispensing (β: −0.18, SE: 0.25, t: −0.71, df: 93, *p*-value: 0.4813). The total pharmacy costs for drug purchase correlated to the percentages of psychotropic drug (β: 0.01, SE: 0.0003, t: 16.80, df: 16,000, *p*-value < 0.0001) (Table 4). On the other hand, the results of adrenal cortical hormones indicated that pharmacies with shorter lengths of operation correlate with drug consumption. The pharmacies in exception regions had higher values for adrenal cortical hormones drugs dispensed compared to pharmacies in the program application regions (β: 0.72, SE: 0.07, t: 9.92, df: 93, *p*-value < 0.0001). The total pharmacy costs for drug purchase were inversely related to the percentages of drugs consumed in the adrenal cortical hormone category. Based on the results of regional-level variables, pharmacies which were located in metropolitan regions had inverse associations with less drug consumption (β: −0.04, SE: 0.02, t: −2.07, df: 241, *p*-value = 0.0395) (Table 5). The results for antibiotics had no interesting findings compared with other categories of drugs. Pharmacies with younger pharmacists were more likely to dispense antibiotic drugs. Also, the total pharmacy costs for drug purchase had inverse associations with drug consumption (Table 6).

**Table 3:** Results for multi-level analyses of the associations with percentages of drug consumption in Antipyretic; Analgesic; Anti-inflammatory drugs

Variables	Antipyretic; Analgesic; Anti-inflammatory drugs																			
	Model 1					Model 2					Model 3					Model 4				
	β	SE	t	df	*P*-Value	β	SE	t	df	*P*-Value	β	SE	t	df	*P*-Value	β	SE	t	df	*P*-Value
Intercept	9.51	0.08	120.05	246	<.0001	12.68	0.99	12.80	246	<.0001	9.23	0.27	34.53	241	<.0001	12.26	1.02	12.03	241	<.0001
Pharmacy characteristics																				
Sex of pharmacist																				
Male						0.64	0.13	5.00	243	<.0001						0.52	0.13	4.03	243	<.0001
Female	Ref	-			-	Ref	-			-	Ref	-			-	Ref	-			-
Age of pharmacist (years)																				
≤45						−0.65	0.18	−3.62	722	0.0003						−0.66	0.18	−3.65	722	0.0003
46–55						−0.82	0.18	−4.48	722	<.0001						−0.82	0.18	−4.45	722	<.0001
56–65						−0.62	0.19	−3.26	722	0.0012						−0.59	0.19	−3.07	722	0.0022
≥66	Ref	-			-	Ref	-			-	Ref	-			-	Ref	-			-
Length of operation																				
≤12 months						−1.06	0.20	−5.35	454	<.0001						−1.08	0.20	−5.47	454	<.0001
13–24 months						−0.80	0.20	−3.97	454	<.0001						−0.81	0.20	−4.01	454	<.0001
≥25 months	Ref	-			-	Ref	-			-	Ref	-			-	Ref	-			-
Separation of drug prescribing and dispensing																				
Exception						3.41	0.82	4.14	93	<.0001						3.19	0.82	3.88	93	0.0002
Application	Ref	-			-	Ref	-			-	Ref	-			-	Ref	-			-
Period of exclusion for reformed program																				
≤18 months						−1.05	0.98	−1.07	88	0.2860						−0.93	0.98	−0.95	88	0.3455
≥19 months	Ref	-			-	Ref	-			-	Ref	-			-	Ref	-			-
Total drug purchase (10 million KRW)						−0.02	0.00	−25.37	16,000	<.0001						−0.02	0.00	−25.93	16,000	<.0001
Regional characteristics																				
Region																				
Metropolitan											−0.53	0.16	−3.22	241	0.0015	−0.56	0.16	−3.57	241	0.0004
Non-metropolitan	Ref	-			-	Ref	-			-	Ref	-			-	Ref	-			-
Total number of clinics in regions with pharmacies																				
≤60											0.21	0.21	1.03	241	0.3027	0.12	0.20	0.61	241	0.5431
≥61	Ref	-			-	Ref	-			-	Ref	-			-	Ref	-			-
Total number of pharmacy in regions with pharmacies																				
≤45											−0.18	0.19	−0.92	241	0.3597	−0.17	0.18	−0.92	241	0.3574
≥46	Ref	-			-	Ref	-			-	Ref	-			-	Ref	-			-
Average of individual income in regions with pharmacies																				
≤38 million KRW											0.47	0.17	2.73	241	0.0067	0.43	0.16	2.63	241	0.0091
≥39 million KRW	Ref	-			-	Ref	-			-	Ref	-			-	Ref	-			-
Proportion of national basic livelihood security beneficiaries in regions with pharmacies											0.07	0.06	1.21	241	0.2271	0.14	0.05	2.72	241	0.0070
Random part^a^	Variance	SE	Z		*P*-Value	Variance	SE	Z		*P*-Value	Variance	SE	Z		*P*-Value	Variance	SD	Z		*P*-Value
Variance of the intercept at the regional level	0.39	0.12	3.26		0.0005	0.35	0.11	3.26		0.0006	0.27	0.10	2.55		0.0054	0.20	0.09	2.29		0.0111
Variance of the intercept at the pharmacy level	65.48	0.73	90.21		<.0001	61.92	0.69	90.23		<.0001	65.44	0.73	90.24		<.0001	61.85	0.69	90.28		<.0001
ICC	0.0059					0.0055					0.0040					0.0032				

Note. The results of multilevel linear regression analysis using mixed model to examine associations between program designation (i.e., exception region or application region) and percentages of drug consumption (antipyretic, analgesic, anti-inflammatory drugs, and psychotropic drugs, adrenal cortical hormones, and antibiotics) in hierarchical data which was consisted of pharmacy and regional levels. Significant level *P <* 0.05. If these values were lower than 0.05, it indicated that there were statistically significant associations between independent variable and drug consumption

Model 1 = empty model, Model 2 = only adjusted for pharmacy-level variables, Model 3 = only adjusted regional-level variables, Model 4 = fully adjusted

*KRW* Republic of Korea Won, *ICC* Intra-class Correlation Coefficient, the results were rounded to the second digit after the decimal point, *df* degrees of freedom

^a^If *p*-value were lower than 0.05, it indicated that each level variable had statistically significant association with the outcome variables. The ICC was defined that the ratio of the between cluster variance to the total variance. It was interpreted as the correlation among observations within the same cluster

**Table 4:** Results for multi-level analyses of the associations with percentages of drug consumption in Psychotropic drugs

Variables	Psychotropic drugs																			
	Model 1					Model 2					Model 3					Model 4				
	β	SE	t	df	*P*-Value	β	SE	t	df	*P*-Value	β	SE	t	df	*P*-Value	β	SE	t	df	*P*-Value
Intercept	0.86	0.02	36.54	246	<.0001	0.38	0.30	1.27	246	0.2040	0.89	0.09	10.24	241	<.0001	0.43	0.31	1.40	241	0.1635
Pharmacists and pharmacy characteristics																				
Sex of pharmacist																				
Male						−0.07	0.04	−1.73	243	0.0842						−0.07	0.04	−1.65	243	0.0995
Female	Ref	-			-	Ref	-			-	Ref	-			-	Ref	-			-
Age of pharmacist (years)																				
≤45						0.15	0.05	2.74	722	0.0064						0.15	0.05	2.70	722	0.0070
46–55						0.16	0.06	2.94	722	0.0034						0.16	0.06	2.88	722	0.0040
56–65						0.14	0.06	2.46	722	0.0142						0.14	0.06	2.42	722	0.0157
≥66	Ref	-			-	Ref	-			-	Ref	-			-	Ref	-			-
Length of operation																				
≤12 months						0.41	0.06	6.88	454	<.0001						0.41	0.06	6.87	454	<.0001
13-24 months						0.22	0.06	3.63	454	0.0003						0.22	0.06	3.62	454	0.0003
≥25 months	Ref	-			-	Ref	-			-	Ref	-			-	Ref	-			-
Separation of drug prescribing and dispensing																				
Exception						−0.18	0.25	−0.72	93	0.4741						−0.18	0.25	−0.71	93	0.4813
Application	Ref	-			-	Ref	-			-	Ref	-			-	Ref	-			-
Period of exclusion for reformed program																				
≤18 months						−0.03	0.30	−0.11	88	0.9134						−0.03	0.30	−0.12	88	0.9075
≥19 months	Ref	-			-	Ref	-			-	Ref	-			-	Ref	-			-
Total drug purchase (10 million KRW)						0.00	0.00	16.85	16,000	<.0001						0.01	0.00	16.80	16,000	<.0001
Regional characteristics																				
Region																				
Metropolitan											−0.05	0.05	−0.90	241	0.3677	−0.02	0.05	−0.34	241	0.7348
Non-metropolitan	Ref	-			-	Ref	-			-	Ref	-			-	Ref	-			-
Total number of clinics in regions with pharmacies																				
≤60											−0.08	0.07	−1.18	241	0.2392	−0.07	0.07	−1.10	241	0.2733
≥61	Ref	-			-	Ref	-			-	Ref	-			-	Ref	-			-
Total number of pharmacy in regions with pharmacies																				
≤45											0.04	0.06	0.63	241	0.5302	0.03	0.06	0.50	241	0.6175
≥46	Ref	-			-	Ref	-			-	Ref	-			-	Ref	-			-
Average of individual income in regions with pharmacies																				
≤38 million KRW											−0.01	0.06	−0.14	241	0.8855	−0.01	0.05	−0.16	241	0.8746
≥39 million KRW	Ref	-			-	Ref	-			-	Ref	-			-	Ref	-			-
Proportion of national basic livelihood security beneficiaries in regions with pharmacies											0.01	0.02	0.75	241	0.4500	0.00	0.02	−0.06	241	0.9499
Random part^a^	Variance	SE	Z		*P*-Value	Variance	SE	Z		*P*-Value	Variance	SE	Z		*P*-Value	Variance	SD	Z		*P*-Value
Variance of the intercept at the regional level	0.04	0.01	3.40		0.0003	0.03	0.01	3.24		0.0006	0.04	0.01	3.45		0.0003	0.03	0.01	3.29		0.0005
Variance of the intercept at the pharmacy level	5.76	0.06	90.23		<.0001	5.64	0.06	90.20		<.0001	5.76	0.06	90.22		<.0001	5.64	0.06	90.19		<.0001
ICC	0.0061					0.0057					0.0064					0.0061				

Note. The results of multilevel linear regression analysis using mixed model to examine associations between program designation (i.e., exception region or application region) and percentages of drug consumption (antipyretic, analgesic, anti-inflammatory drugs, and psychotropic drugs, adrenal cortical hormones, and antibiotics) in hierarchical data which was consisted of pharmacy and regional levels. Significant level *P <* 0.05. If these values were lower than 0.05, it indicated that there were statistically significant associations between independent variable and drug consumption

Model 1 empty model, Model 2 only adjusted for pharmacy-level variables, Model 3 only adjusted regional-level variables, Model 4 fully adjusted

*KRW* Republic of Korea Won, *ICC* Intra-class Correlation Coefficient, the results were rounded to the second digit after the decimal point, *df* degrees of freedom

^a^If *p*-value were lower than 0.05, it indicated that each level variable had statistically significant association with the outcome variables. The ICC was defined that the ratio of the between cluster variance to the total variance. It was interpreted as the correlation among observations within the same cluster

**Table 5:** Results for multi-level analyses of the associations with percentages of drug consumption in Adrenal cortical hormones

Variables	Adrenal cortical hormones																			
	Model 1					Model 2					Model 3					Model 4				
	β	SE	t	df	*P*-Value	β	SE	t	df	*P*-Value	β	SE	t	df	*P*-Value	β	SE	t	df	*P*-Value
Intercept	0.30	0.01	32.20	246	<.0001	0.38	0.09	4.38	246	<.0001	0.31	0.03	9.15	241	<.0001	0.41	0.09	4.48	241	<.0001
Pharmacists and pharmacy characteristics																				
Sex of pharmacist																				
Male						0.00	0.01	−0.02	243	0.9876						0.00	0.01	−0.19	243	0.8498
Female	Ref	-			-	Ref	-			-	Ref	-			-	Ref	-			-
Age of pharmacist (years)																				
≤45						0.04	0.02	2.25	722	0.0251						0.03	0.02	2.10	722	0.0361
46–55						0.00	0.02	−0.21	722	0.8342						−0.01	0.02	−0.37	722	0.7139
56–65						−0.02	0.02	−0.93	722	0.3528						−0.02	0.02	−1.01	722	0.3152
≥66	Ref	-			-	Ref	-			-	Ref	-			-	Ref	-			-
Length of operation																				
≤12 months						0.05	0.02	2.78	454	0.0057						0.05	0.02	2.74	454	0.0064
13–24 months						0.03	0.02	1.92	454	0.0555						0.03	0.02	1.89	454	0.0595
≥25 months	Ref	-			-	Ref	-			-	Ref	-			-	Ref	-			-
Separation of drug prescribing and dispensing																				
Exception						0.72	0.07	10.05	93	<.0001						0.72	0.07	9.92	93	<.0001
Application	Ref	-			-	Ref	-			-	Ref	-			-	Ref	-			-
Period of exclusion for reformed program																				
≤18 months						−0.10	0.09	−1.16	88	0.2484						−0.10	0.09	−1.17	88	0.2442
≥19 months	Ref	-			-	Ref	-			-	Ref	-			-	Ref	-			-
Total drug purchase (10 million KRW)						0.00	0.00	−3.68	16,000	0.0002						0.00	0.00	−3.68	16,000	0.0002
Regional characteristics																				
Region																				
Metropolitan											−0.06	0.02	−3.00	241	0.0030	−0.04	0.02	−2.07	241	0.0395
Non-metropolitan	Ref	-			-	Ref	-			-	Ref	-			-	Ref	-			-
Total number of clinics in regions with pharmacies																				
≤60											0.01	0.03	0.24	241	0.8108	−0.01	0.02	−0.27	241	0.7887
≥61	Ref	-			-	Ref	-			-	Ref	-			-	Ref	-			-
Total number of pharmacy in regions with pharmacies																				
≤45											0.02	0.02	0.98	241	0.3303	0.01	0.02	0.50	241	0.6149
≥46	Ref	-			-	Ref	-			-	Ref	-			-	Ref	-			-
Average of individual income in regions with pharmacies																				
≤38 million KRW											0.01	0.02	0.37	241	0.7088	−0.01	0.02	−0.31	241	0.7555
≥39 million KRW	Ref	-			-	Ref	-			-	Ref	-			-	Ref	-			-
Proportion of national basic livelihood security beneficiaries in regions with pharmacies											0.00	0.01	−0.72	241	0.4740	0.00	0.01	−0.52	241	0.6035
Random part^a^	Variance	SE	Z		*P*-Value	Variance	SE	Z		*P*-Value	Variance	SE	Z		*P*-Value	Variance	SD	Z		*P*-Value
Variance of the intercept at the regional level	0.01	0.00	4.76		<.0001	0.01	0.00	4.13		<.0001	0.01	0.00	4.42		<.0001	0.01	0.00	3.99		<.0001
Variance of the intercept at the pharmacy level	0.48	0.01	89.74		<.0001	0.47	0.01	89.77		<.0001	0.48	0.01	89.72		<.0001	0.47	0.01	89.76		<.0001
ICC	0.0223					0.0151					0.0199					0.0149				

Note. The results of multilevel linear regression analysis using mixed model to examine associations between program designation (i.e., exception region or application region) and percentages of drug consumption (antipyretic, analgesic, anti-inflammatory drugs, and psychotropic drugs, adrenal cortical hormones, and antibiotics) in hierarchical data which was consisted of pharmacy and regional levels. Significant level *P <* 0.05. If these values were lower than 0.05, it indicated that there were statistically significant associations between independent variable and drug consumption

Model 1 = empty model, Model 2 = only adjusted for pharmacy-level variables, Model 3 = only adjusted regional-level variables, Model 4 = fully adjusted

*KRW* Republic of Korea Won, *ICC* Intra-class Correlation Coefficient, the results were rounded to the second digit after the decimal point. *df* degrees of freedom

^a^If *p*-value were lower than 0.05, it indicated that each level variable had statistically significant association with the outcome variables. The ICC was defined that the ratio of the between cluster variance to the total variance. It was interpreted as the correlation among observations within the same cluster

**Table 6:** Results for multi-level analyses of the associations with percentages of drug consumption in Antibiotics

Variables	Antibiotics																			
	Model 1					Model 2					Model 3					Model 4				
	β	SE	t	df	*P*-Value	β	SE	t	df	*P*-Value	β	SE	t	df	*P*-Value	β	SE	t	df	*P*-Value
Intercept	7.00	0.18	39.29	246	<.0001	6.56	2.83	2.31	246	0.0216	7.72	0.62	12.51	241	<.0001	7.29	2.90	2.52	241	0.0125
Pharmacists and pharmacy characteristics																				
Sex of pharmacist																				
Male						0.08	0.36	0.23	243	0.8207						0.17	0.37	0.45	243	0.6551
Female	Ref	-			-	Ref	-			-	Ref	-			-	Ref	-			-
Age of pharmacist (years)																				
≤45						3.49	0.51	6.78	722	<.0001						3.42	0.52	6.58	722	<.0001
46–55						2.35	0.52	4.49	722	<.0001						2.24	0.53	4.25	722	<.0001
56–65						1.22	0.55	2.24	722	0.0255						1.12	0.55	2.05	722	0.0404
≥66	Ref	-			-	Ref	-			-	Ref	-			-	Ref	-			-
Length of operation																				
≤12 months						2.02	0.57	3.58	454	0.0004						2.02	0.57	3.57	454	0.0004
13–24 months						0.27	0.58	0.47	454	0.6417						0.25	0.58	0.44	454	0.6632
≥25 months	Ref	-			-	Ref	-			-	Ref	-			-	Ref	-			-
Separation of drug prescribing and dispensing																				
Exception						−1.49	2.35	−0.63	93	0.5283						−1.55	2.35	−0.66	93	0.5134
Application	Ref	-			-	Ref	-			-	Ref	-			-	Ref	-			-
Period of exclusion for reformed program																				
≤18 months						−0.27	2.81	−0.10	88	0.9240						−0.45	2.81	−0.16	88	0.8720
≥19 months	Ref	-			-	Ref	-			-	Ref	-			-	Ref	-			-
Total drug purchase (10 million KRW)						−0.02	0.00	−7.93	16,000	<.0001						−0.02	0.00	−7.63	16,000	<.0001
Regional characteristics																				
Region																				
Metropolitan											−0.03	0.39	−0.07	241	0.9466	−0.02	0.39	−0.04	241	0.9659
Non-metropolitan	Ref	-			-	Ref	-			-	Ref	-			-	Ref	-			-
Total number of clinics in regions with pharmacies																				
≤60											−0.31	0.49	−0.62	241	0.5331	−0.30	0.49	−0.61	241	0.5409
≥61	Ref	-			-	Ref	-			-	Ref	-			-	Ref	-			-
Total number of pharmacy in regions with pharmacies																				
≤45											0.85	0.46	1.86	241	0.0646	0.55	0.46	1.21	241	0.2270
≥46	Ref	-			-	Ref	-			-	Ref	-			-	Ref	-			-
Average of individual income in regions with pharmacies																				
≤38 million KRW											−0.54	0.41	−1.31	241	0.1917	−0.30	0.41	−0.75	241	0.4568
≥39 million KRW	Ref	-			-	Ref	-			-	Ref	-			-	Ref	-			-
Proportion of national basic livelihood security beneficiaries in regions with pharmacies											−0.26	0.14	−1.94	241	0.0537	−0.19	0.14	−1.42	241	0.1584
Random part^a^	Variance	SE	Z		*P*-Value	Variance	SE	Z		*P*-Value	Variance	SE	Z		*P*-Value	Variance	SD	Z		*P*-Value
Variance of the intercept at the regional level	0.06	0.40	0.15		0.4411	4.02	33759544.00	0.00		0.5000	0.10	0.39	0.25		0.4014	0.05	0.38	0.14		0.4462
Variance of the intercept at the pharmacy level	516.25	5.71	90.49		<.0001	511.21	5.64	90.68		<.0001	515.85	5.70	90.50		<.0001	511.09	5.65	90.48		<.0001
ICC	0.0001					0.0078					0.0002					0.0001				

Note. The results of multilevel linear regression analysis using mixed model to examine associations between program designation (i.e., exception region or application region) and percentages of drug consumption (antipyretic, analgesic, anti-inflammatory drugs, and psychotropic drugs, adrenal cortical hormones, and antibiotics) in hierarchical data which was consisted of pharmacy and regional levels. Significant level *P <* 0.05. If these values were lower than 0.05, it indicated that there were statistically significant associations between independent variable and drug consumption

Model 1 = empty model, Model 2 = only adjusted for pharmacy-level variables, Model 3 = only adjusted regional-level variables, Model 4 = fully adjusted

*KRW* Republic of Korea Won, *ICC* Intra-class Correlation Coefficient, the results were rounded to the second digit after the decimal point, *df* degrees of freedom

^a^If *p*-value were lower than 0.05, it indicated that each level variable had statistically significant association with the outcome variables. The ICC was defined that the ratio of the between cluster variance to the total variance. It was interpreted as the correlation among observations within the same cluster

Using the methodologies related to multilevel linear regression analysis, we investigated model specification by comparing the results in each model (model 1 to 4). Based on the results, it can be seen that both pharmacy—and regional-level variables had statistically significant associated to outcome variables, but that there were no significant relationship regarding antibiotics. These results showed that regional characteristics do not have significant associations in the consumption of antibiotics (Table 6). We compared the ICC to examine the reliability of each level variable for the outcome variable. The ICC in the fully adjusted model were estimated as follows = Antipyretic; Analgesic; Anti-inflammatory drugs: 0.0032, Psychotropic drugs: 0.0061, Adrenal cortical hormones: 0.0149, and Antibiotics: 0.0001. These values indicated that the regional-level variables explained the 0.3, 0.6, 1.5, and 0.01 % of the total variability in outcomes, respectively (Tables 3, 4, 5 and 6).

**Fig. 2:**
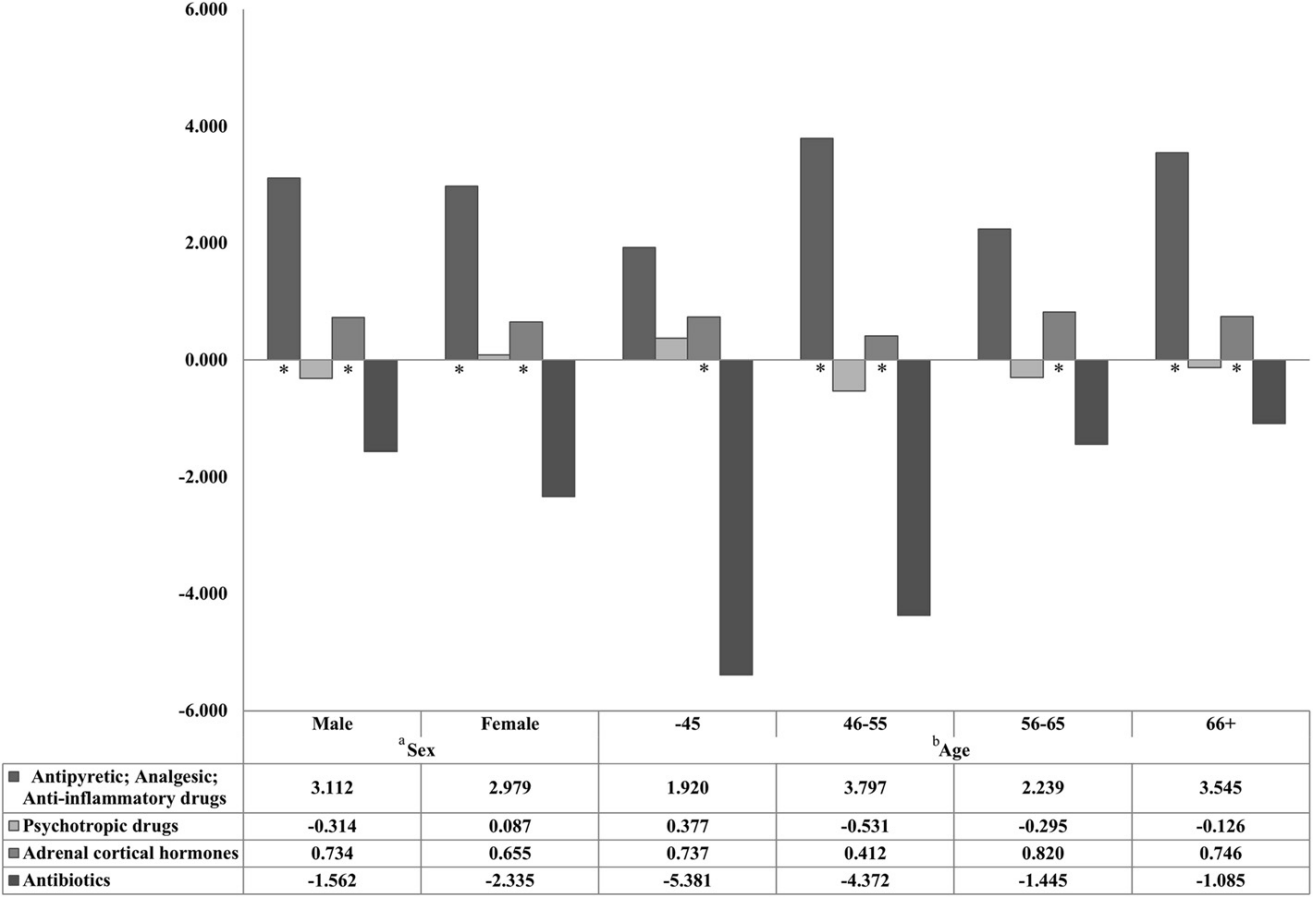
Results of the sub-group analysis on the relationships between drug prescribing and dispensing exception and application regions and drug consumption, by sex and age of the pharmacists. *Statistically significant difference, multilevel linear regression analysis using mixed model. ^a^The results of the sub-group analysis by sex of pharmacist, (Male) Antipyretic; Analgesic; Anti-inflammatory drugs = t: 2.76, degrees of freedom (df): 80, *p*-value: 0.0071; Psychotropic drugs = t: −1.11, df: 80, *p*-value: 0.2721; Adrenal cortical hormones = t: 8.60, df: 80, *p*-value <0.0001; Antibiotics = t:-0.42, df: 80, *p*-value: 0.6789. (Female) Antipyretic; Analgesic; Anti-inflammatory drugs = t: 2.36, df: 48, *p*-value: 0.0224; Psychotropic drugs = t: 0.18, df: 48, *p*-value: 0.8608; Adrenal cortical hormones = t: 4.77, df: 48, *p*-value < 0.0001; Antibiotics = t: −1.37, df: 48, *p*-value: 0.1756. ^b^The results of the sub-group analysis by age of pharmacist, (Less than 45 years) Antipyretic; Analgesic; Anti-inflammatory drugs = t: 0.49, df: 21, *p*-value: 0.6292; Psychotropic drugs = t: 0.41, df: 21, *p*-value: 0.6878; Adrenal cortical hormones = t: 2.87, df: 21, *p*-value: 0.0092; Antibiotics = t: −0.46, df: 21, *p*-value: 0.6475. (46–55 years) Antipyretic; Analgesic; Anti-inflammatory drugs = t: 2.44, df: 37, *p*-value: 0.0194; Psychotropic drugs = t: −0.85, df: 37, *p*-value: 0.4020; Adrenal cortical hormones = t: 2.78, df: 37, *p*-value: 0.0085; Antibiotics = t: −2.11, df: 37, *p*-value: 0.0415. (56–65 years) Antipyretic; Analgesic; Anti-inflammatory drugs = t: 1.80, df: 48, *p*-value < 0.001; Psychotropic drugs = t: −0.50, df: 48, *p*-value: 0.6207; Adrenal cortical hormones = t: 6.26, df: 48, *p*-value < .0001; Antibiotics = t: −0.72, df: 48, *p*-value: 0.4739. (More than 65 years) Antipyretic; Analgesic; Anti-inflammatory drugs = t: 3.70, df: 63, *p*-value: 0.0005; Psychotropic drugs = t: −0.51, df: 63, *p*-value: 0.6134; Adrenal cortical hormones = t: 6.05, df: 63, *p*-value < 0.0001; Antibiotics = t: −0.29, df: 63, *p*-value: 0.7746

**Fig. 3:**
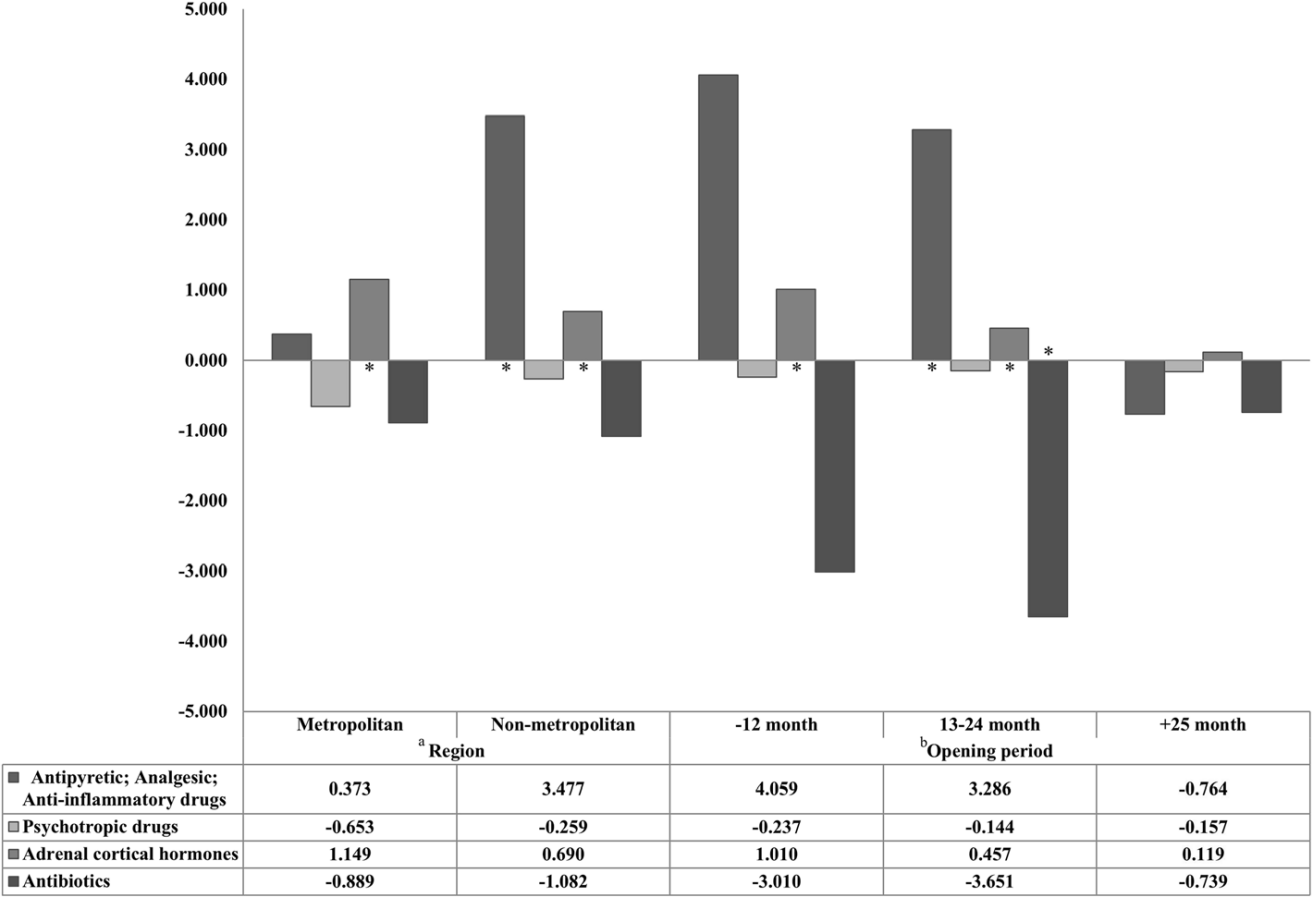
Results of the sub-group analysis on the relationships between drug prescribing and dispensing exception regions and drug consumption, by region and time since pharmacy operation. *Statistically significant difference, multilevel linear regression analysis using mixed model. ^a^The results of the sub-group analysis by pharmacy region, (Metropolitan) Antipyretic; Analgesic; Anti-inflammatory drugs = t: 0.11, df: 6, *p*-value: 0.9173; Psychotropic drugs = t: −0.44, df: 6, *p*-value: 0.6755; Adrenal cortical hormones = t: 3.17, df: 6, *p*-value: 0.0194; Antibiotics = t: −0.08, df: 6, *p*-value: 0.9388. (Non-metropolitan) Antipyretic; Analgesic; Anti-inflammatory drugs = t: 3.47, df: 86, *p*-value: 0.0008; Psychotropic drugs = t: −1.11, df: 86, *p*-value: 0.2710; Adrenal cortical hormones = t: 8.67, df: 86, *p*-value < 0.0001; Antibiotics = t: −0.40, df: 86, *p*-value: 0.6922. ^b^The results of the sub-group analysis by time since pharmacy operation, (Less than 12 months) Antipyretic; Analgesic; Anti-inflammatory drugs = t: 1.68, df: 23, *p*-value: 0.1061; Psychotropic drugs = t: −0.58, df: 23, *p*-value: 0.5694; Adrenal cortical hormones = t: 6.85, df: 23, *p*-value < 0.0001; Antibiotics = t: −0.35, df: 23, *p*-value: 0.7317. (13–24 months) Antipyretic; Analgesic; Anti-inflammatory drugs = t: 3.08, df: 25, *p*-value: 0.0050; Psychotropic drugs = t: −0.40, df: 25, *p*-value: 0.6932; Adrenal cortical hormones = t: 3.31, df: 25, *p*-value: 0.0029; Antibiotics = t: −2.49, df: 25, *p*-value: 0.0196. (More than 25 months) Antipyretic; Analgesic; Anti-inflammatory drugs = t: −0.56, df: 86, *p*-value: 0.5798; Psychotropic drugs = t: −0.26, df: 86, *p*-value: 0.7928; Adrenal cortical hormones = t: 0.79, df: 86, *p*-value: 0.4337; Antibiotics = t: −0.40, df: 86, *p*-value: 0.6922

We also performed sub-group analyses for multilevel regression analysis in exception regions by the sex of pharmacist, age of pharmacist, regional characteristics of the pharmacy, and the length of time the pharmacy had been in operation. The results for the sub-group analysis by sex indicated that for both sex groups, exception regions had higher percentages of drug consumption of drugs in the antipyretic, analgesic, anti-inflammatory category, and for adrenal cortical hormones category, compared to the application regions. The results of the sub-group analysis performed by age was generally similar to the results by sex, but the percent of antibiotics consumption for the 46–55 year age group was higher in the application regions (Fig. 2). The results regarding the regional characteristics of the pharmacies revealed that in non-metropolitan regions, exception region pharmacies had higher consumption percentages of antipyretic, analgesic, anti-inflammatory drugs, in addition to adrenal cortical hormones, compared with application region pharmacies. Regarding the operation time of pharmacies, the values for the antipyretic, analgesic, anti-inflammatory drugs category and the adrenal cortical hormones category were higher for the exception region pharmacies, especially for pharmacies that had been in operation for 13–24 months (Fig. 3).

## Discussion

Access and consumption of health care have substantially increased as socio-economic conditions have improved in South Korea. This increase has resulted in new problems, such as increasing medical costs, increased disease cost-burdens, and increased misuse of drugs. To solve these problems, the South Korean government introduced a program that separated drug prescribing from dispensing [15, 16]. Many articles describing the effects of this program have been published by health care professionals since the program was introduced. Compared with other OECD countries, drug consumption in South Korea has continued to increase, but few investigators have examined drug consumption characteristics in the program-designated exception regions [6]. We therefore analyzed the relationships between exception/application region characteristics and the four categories of drug consumption to investigate how various factors affect drug consumption.

The results of our study indicated that compared with the regions designated as program application regions, exception regions had higher values for percent consumption of drugs in the antipyretic, analgesic, anti-inflammatory drugs category, and in the adrenal cortical hormones category. It suggested that different prescription pattern in pharmacies by whether designation of program for separated drug prescribing from dispensing. We also performed sub-group analyses by pharmacist (i.e., sex, age) and pharmacy (e.g., region, time since pharmacy began operation) characteristics. The exception region male pharmacists had higher values for percent consumption of antipyretic, analgesic, anti-inflammatory drugs and for adrenal cortical hormones, compared with female pharmacists. Exception region pharmacies in non-metropolitan regions also had higher values for percent consumption of antipyretic, analgesic, anti-inflammatory drugs, compared with application pharmacies in non-metropolitan regions.

Our findings suggest that the government should consider the characteristics of pharmacies and pharmacists when managing misuse of drugs because different prescription patterns in pharmacy with region in exception of program for separated drug prescribing from dispensing could cause misuse behavior in patients. Some alternative approaches and regulations arise from our findings. First, under the existing legislation, pharmacists in exception regions may dispense drugs (including “prescription only medicine”) for less than 5 days without a doctor’s prescription. However, in cases outlined by the Korea Food & Drug Administration, some drugs for which there are concerns about misuse have been designated as drugs only prescribed by doctors [17]. Therefore, drugs at a high risk for adverse events should be placed in a special category by the government. Second, it is difficult to monitor drug consumption in exception regions. Thus, an efficient monitoring system should be established that includes exception regions and application regions. These monitoring systems could help to prevent drug misuse [18–21]. Third, an education program that that targets patients at risk for drug misuse could help to prevent excessive spending. The results of previous studies indicate that drug use education programs can effectively assist patients [22, 23]. These additional activities by the government could be helpful for reducing unconscious misuse [24]. Finally, based on our results, there were different patterns of prescriptions by total drug purchase amount as indirect indicator for size of each pharmacy. Also, by the categories of drugs, there were different patterns of drug consumption. Thus, it is needed to consider different management strategies by considering the size of pharmacy and types of drugs [25].

This study had some strengths compared to previous investigations. First, to our knowledge, this is first report on the relationships between the consumption of drugs at risk for misuse and pharmacy and regional-related factors in program exception compared with program application regions. Previous studies of drug consumption only included program application regions. Therefore, our results will be useful to health policy makers and professionals for the management of drug consumption. Our study results are also useful for the management of drug consumption in South Korea because we investigated both drugs covered by the National Health Insurance Services (NHIs) and drugs not covered by the NHIs. Most of the previous studies examined only data related to NHIs drugs [15, 26]. Thus, our results are an indication of the overall status of drug consumption in South Korea. Our findings can be used by health policy makers to develop efficient alternative policies.

Our study also had some limitations. First, there might be different medical needs for different population in each pharmacy. However, we could not consider drug consumption details for each prescription or each patient’s case, because we only included the pharmacy—and regional-related factors due to limitations of data. We also could not analyze the compliance rate of each patient due to such limitations, even though compliance rate was important factors in analyzing the pharmaceutical expenditures. Next, we also did not examine relationships at scale, because our dataset did not include information on the total drug stock inventory at the beginning of the study period. Third, we only considered four categories of drugs. This is because those drugs were reported to cause many side effects by Korean Food and Drugs Administration. In addition, by the previous studies, misuse for those drugs could cause fatal results such as admission in patients with chronic diseases [27, 28]. Fourth, in South Korea, there are 26,063 pharmacies during 2011 to 2013. However, the prescription pattern of each pharmacy could affect by characteristics of each pharmacy such as number of pharmacists. To analyze with reducing the variation of prescription pattern, we only included pharmacies with one pharmacist (*N =* 16,455; 63.1 % among overall South Korea) in our dataset. For that reasons, there were some limitations that it would be difficult to generalize to the overall South Korean population. Fifth, the length of the study period was a bit short to reveal the effects of introducing the program. The beginning of the study period was >10 years after the date that the program was introduced. Finally, we only used to ratio of purchase about for four categories drugs as outcomes variables to investigate the difference patterns of prescription by designation of program. It was best way if we could use other types of indicators. However, we could not consider those due to limitation of data.

Despite these limitations, our results suggested that compared with pharmacies in program application regions, pharmacies in program exception regions were more likely to supply antipyretic, analgesic, anti-inflammatory drugs, and adrenal cortical hormones. Based on these results, health care professionals and health policy makers should consider managing health care expenditures by categories of drugs consumed, especially in exception regions. More detailed studies of prescribing patterns that use datasets larger than our dataset are needed to determine effective strategies.

## Conclusions

Compared with program-designated application regions, drug consumption of antipyretic, analgesic, anti-inflammatory drugs, and of adrenal cortical hormones was higher in the program regions designated as exception regions to the rule that separates drug prescribing and dispensing. Characteristics of both pharmacists and pharmacies are associated with drug consumption patterns in program exception regions.

## Abbreviation

OECD: Organization for Economic Co-operation and Development
GDP: Gross Domestic Product
SD: Standard Deviation
SE: Standard Error
ICC: Intra-class Correlation Coefficient
df: Degrees of freedom
NHIs: National Health Insurance Services
KRW: Republic of Korea Won
Ref: Reference
